# Characterizing hypertension among recreational cyclists in Colombia: The Atlántico cyclists study

**DOI:** 10.1016/j.pmedr.2025.103105

**Published:** 2025-05-10

**Authors:** Bryan Hernandez Nieto, Manuel Urina-Jassir, Mirary Mantilla-Morron, Carolina Rosa Charris Cogollo, Riguey Mercado Marchena, Daniela Urina-Jassir, Manuel Urina-Triana, Miguel Urina-Triana

**Affiliations:** a Faculty of Health Sciences, Universidad Simón Bolívar, Barranquilla, Colombia; b Department of Medicine, Boston Medical Center and Boston University Chobanian and Avedisian School of Medicine, Boston, MA, United States; c Columbia University Division of Cardiology at Mount Sinai Medical Center, Miami Beach, FL, United States.; d Department of Clinical Research, Fundación del Caribe para la Investigación Biomédica, Barranquilla, Colombia; e Life Sciences Research Center, Universidad Simón Bolívar, Barranquilla, Colombia

**Keywords:** Exercise, Bicycling, Hypertension, Cardiometabolic risk factors, Latin America

## Abstract

**Objective:**

To describe the prevalence of hypertension and exercise-induced hypertension (EIH) among recreational cyclists in addition to their exercise habits and prior medical evaluations.

**Methods:**

A cross-sectional study was conducted among adult recreational cyclists from March to April 2024 in the Department of Atlántico, Colombia. Participants were selected via convenience sampling after cycling activities and data were collected with a structured survey (demographics, comorbidities, and exercise habits). Post-exercise blood pressure (BP; two measurements three minutes apart), heart rate, oxygen saturation, weight, height, and waist circumference were measured. EIH was defined as a systolic BP > 210 mmHg for men and > 190 mmHg for women. Data were summarized with descriptive statistics**.**

**Results:**

Three hundred and fifty-five individuals were included. Most were male (84.5 %) and older than 45 years of age (75.8 %). Hypertension was identified in 22 % of participants. Other risk factors included hypercholesterolemia (11.8 %), smoking (10.4 %), and hypertriglyceridemia (7.3 %). The mean post-exercise systolic BPs were 130.4 ± 55.6 mmHg and 122.6 ± 15.5 mmHg, and diastolic BPs were 77.2 ± 10.4 mmHg and 76.8 ± 10.7 mmHg (initial and three minutes later, respectively) and no participants fulfilled the criteria for EIH. Among the participants, 20.6 % underwent prior medical evaluation.

**Conclusions:**

Hypertension was a common baseline condition among recreational cyclists, but none was found to have EIH. Despite a high prevalence of cardiovascular risk factors, one-fifth of the participants had a medical evaluation before engaging in cycling activities. Our findings underscore the importance of encouraging routine health screenings in this population.

## 1 Introduction

Hypertension (HTN) is a major risk factor for cardiovascular diseases (CVDs) and CVD-related mortality (Rethy et al., 2020). Regular physical activity is widely recommended due to its cardiovascular benefits and role in the prevention of chronic conditions such as HTN (Edwards et al., 2023). However, HTN is one of the most frequent comorbidities identified in athletes, with prevalence ranging between 0 % and 83 % in a systematic review (Berge et al., 2015). Most recently, authors have described a phenomenon called exercise-induced hypertension (EIH), or hypertensive response to exercise (HRE), characterized by an increase in systolic blood pressure (SBP) during or immediately after exercise. Exercise-induced hypertension is defined by a SBP > 210 mmHg in men and > 190 mmHg in women during exercise (Mohammed et al., 2020; Schultz and Sharman, 2014). It has been recognized as a risk factor for future HTN and cardiac remodeling, as well as a potential risk factor for CVDs and sudden cardiac death (Ghekiere et al., 2023; Kim and Park, 2024). Exercise-induced hypertension can be identified in healthy subjects without cardiovascular risk factors (Mohammed et al., 2020). Thus, the identification of EIH and its accompanying cardiovascular risk merits being studied.

Cycling, a popular form of dynamic and aerobic exercise, has been associated with lower all-cause and CVD mortality risk in patients with diabetes (compared to non-cyclists) and with a reduction in hemoglobin A1c, blood pressure (BP), and weight (Jhingan and Jhingan, 2017; Ried-Larsen et al., 2021). Furthermore, the UK Cycling for Health Study describes a dose-response association between cycling volume and the risk of HTN, with higher categories of activity being associated with a lower likelihood of HTN and hypercholesterolemia (Hollingworth et al., 2015). However, data on the prevalence of HTN and EIH in recreational cyclists is lacking.

Identifying cardiovascular risk factors and EIH in these individuals is crucial to developing specific interventions aimed at mitigating cardiovascular risk while promoting the benefits of physical activity. Thus, this study aimed to characterize the prevalence of HTN and EIH among recreational cyclists in the Department of Atlántico, Colombia. Moreover, we sought to assess their prior comorbidities and the frequency of prior medical evaluations.

## 2 Methods

This study is reported following The Strengthening the Reporting of Observational Studies in Epidemiology (STROBE) statement (von Elm et al., 2008). We followed the Declaration of Helsinki, and the Ethics Committee of Fundación del Caribe para la Investigación Biomédica (Acta 296, February 29th, 2024) approved the study. All patients provided informed consent.

### 2.1 Study design and setting

We conducted a cross-sectional observational study among recreational cyclists in the Department of Atlántico (North Caribbean Region) in Colombia from March 2024 to April 2024.

### 2.2 Participants

The study population consisted of recreational cyclists in the community who were approached at a common location frequented by cyclists in the Department of Atlántico, Colombia. Thus, a non-probabilistic convenience sampling was used. We included individuals who were older than 18 years, practiced recreational cycling regularly, accepted to participate in the study, and provided informed consent. Pre-specified exclusion criteria included incompleteness of the required assessments, such as study survey and vital signs measurement; however, all participants who were approached for the study accepted to participate and completed the measurements.

### 2.3 Data sources and measurement

A structured survey was designed and used for data collection. This survey included demographic data (e.g., age, gender), comorbidities and risk factors, and physical exercise habits tailored to their cycling experience (the frequency and duration of recreational cycling). Regarding baseline comorbidities and risk factors, participants were surveyed on the presence or absence of common cardiovascular conditions and risk factors. For this study, hypertension was defined as self-reported (i.e., affirmative response in the study survey) hypertension.

Blood pressure was measured with a validated automatic BP monitor with an appropriately sized cuff for the subject's arm. Two measurements were obtained immediately after physical activity and three minutes later. Blood pressure was classified as elevated if higher than 140/90 mmHg at rest, and EIH was defined as a systolic BP > 210 mmHg in men and > 190 mmHg in women**.**

Moreover, additional measurements included weight, height, waist circumference, heart rate, and oxygen saturation (using a finger pulse oximeter). As for BP measurement, heart rate was measured after physical activity and subsequently, three minutes later. Waist circumference was considered high if ≥89 cm in men or ≥ 86 cm in women, as recommended in a recent cohort study in a Colombian population (Lopez-Lopez et al., 2023).

### 2.4 Statistical analysis

Descriptive statistics were used to summarize the findings. Categorical variables are presented as frequency (%) and continuous variables as mean ± standard deviation (SD). Data were analyzed using SPSS statistical software version 26 for Mac.

## 3 Results

A total of 355 recreational cyclists were included. Most participants were men (*n* = 300; 84.5 %), and 75.8 % (*n* = 269) were 45 or older. The most common predisposing conditions were HTN (*n* = 78; 22 %), hypercholesterolemia (*n* = 42; 11.8 %), and hypertriglyceridemia (n = 26; 7.3 %). Moreover, 10.4 % (*n* = 37) reported a history of smoking. The majority of participants (*n* = 159; 44.8 %) had between 1 and 5 years of cycling experience and cycled 3 to 4 days per week (*n* = 163; 45.9 %). On the day of measurement, 46.8 % (*n* = 166) had done more than 90 min of cycling. Furthermore, 20.6 % (*n* = 73) of the participants had completed a medical evaluation before starting their recreational cycling practice **(**Table 1**)**.

**Table 1:** Baseline characteristics, cycling activities, and vital signs/anthropometric measurements among adult recreational cyclists in the Department of Atlántico, Colombia, March 2024 – April 2024 (*n* = 355).

Baseline Characteristics, Exercise Habits, and Measurements		n (%)
*Demographic and baseline characteristics*		
Gender	Male	300 (84.5)
	Female	55 (15.5)
Age groups	18–44	86 (24.2)
	45–64	211(59.4)
	≥65	58 (16.4)
Comorbidities & Risk Factors	Hypertension	78 (22)
	Hypercholesterolemia	42 (11.8)
	Smoking	37 (10.4)
	Hypertriglyceridemia	26 (7.3)
	Type 2 Diabetes Mellitus	19 (5.4)
	Myocardial Infarction	7 (2.0)
	Hypothyroidism	4 (1.1)
*Cycling activities*		
Cycling experience (years)	<1	22 (6.2)
	1–5	159 (44.8)
	6–10	69 (19.4)
	>10	105 (29.6)
Cycling frequency (days per week)	1–2	113 (31.8)
	3–4	163 (45.9)
	>5	79 (22.3)
Cycling time on the day of measurement (minutes)	<30	13 (3.7)
	40–60	96 (27)
	61–90	80 (22.5)
	>90	166 (46.8)
*Anthropometric measures and vital signs*		
BMI (kg/m^2^)	<18	3 (0.8)
	18–24.9	120 (33.8)
	25–29.9	163 (45.9)
	≥30	69 (19.4)
Waist circumference (cms)^a^	Male ≤88	120 (40)
	Male ≥89	180 (60)
	Female ≤85	45 (82)
	Female ≥86	10 (18)
SBP^b^ (mean ± SD, mm Hg)	Initial	130.4 ± 55.6
	Subsequent	122.6 ± 15.5
DBP^b^ (mean ± SD, mm Hg)	Initial	77.2 ± 10.4
	Subsequent	76.8 ± 10.7
Heart Rate^b^ (mean ± SD, bpm)	Initial	98.3 ± 16.1
	Subsequent	95.3 ± 15.2
SpO2^c^ (%)	96.0 ± 4.2	

**Abbreviations:** BMI: body mass index; BPM: beats per minute; SBP: systolic blood pressure; DBP: diastolic blood pressure.

a A high waist circumference in Colombia is defined as ≥86 cm in women and ≥ 89 cm in men.

b The first measurement was obtained immediately after cycling, and the second measurement was obtained 3 min after cycling.

c The measurement of SpO2 (%) was obtained after cycling.

Most participants had a BMI in the range of overweight or obesity (*n* = 232; 65.3 %). Waist circumference was elevated in 60 % (*n* = 180) of men (≥ 89 cm) and 18 % (*n* = 10) of women (≥ 86 cm) **(**Table 1**).** The initial mean SBP immediately after exercise was 130.4 ± 55.6 mmHg, decreasing to 122.6 ± 15.5 mmHg in the subsequent measurement performed 3 min later. Initial mean diastolic BP showed a slight decrease from 77.2 ± 10.4 mmHg to 76.8 ± 10.7 mmHg between the first and second measurements. None of the participants met the criteria for EIH immediately after exercise.

## 4 Discussion

This cross-sectional study provides valuable information on the prevalence of HTN and cardiovascular risk factors in a population of adult recreational cyclists, as well as insight into their exercise habits and prior medical evaluation practices.

Our findings reveal a prevalence of HTN of 22 % among the studied population. Importantly, this prevalence reflects pre-existing conditions and is not directly related to cycling. This prevalence is consistent with the previously reported Colombian national prevalence of HTN, estimated at 24 % in a meta-analysis (Zurique Sánchez et al., 2019). It has been reported that physical activity, including both aerobic and dynamic exercise, has a beneficial effect on BP control (Edwards et al., 2023; Oja et al., 2011). Furthermore, we identified the presence of other cardiovascular risk factors, such as hypercholesterolemia (11.8 %), smoking (10.4 %), and type 2 diabetes mellitus (5.4 %), underscoring the importance of a complete cardiovascular risk assessment of individuals (Franklin et al., 2020). Lastly, the high prevalence of overweight and obesity (65.3 %) among the participants is a significant finding. Although regular cycling can contribute to the reduction of body mass index, it is evident that other factors such as diet, sedentary lifestyle outside of cycling, and genetic and metabolic factors can influence this result (Swift et al., 2014). Overall, this frequency of HTN and other cardiovascular risk factors might indicate that these individuals are seeking lifestyle modifications with recreational cycling.

A relevant finding of our study is the absence of cases of EIH among the participants. The prevalence of EIH can vary significantly depending on the population studied and the criteria used for its definition. For instance, Caselli et al. describe a prevalence of high BP response to exercise (defined as values above the 95th percentile) of 3.2 % for isolated systolic, 4.3 % of isolated diastolic, and 0.5 % of both systolic/diastolic in a large cohort of competitive athletes (Caselli et al., 2016). However, our population of recreational cyclists may differ significantly from competitive athletes in terms of exercise intensity and cardiovascular adaptations. The absence of EIH in our sample could be explained by several reasons. First, the timing of BP measurement (immediately after exercise and 3 min later) did not capture peak BP during exercise. Second, the recreational nature of cycling in our population may involve different exercise intensities compared to competitive sports, and given our study design, we were unable to quantify and standardize the exercise intensity reached by each participant. Lastly, the long-term cardiovascular adaptation in regular cyclists could confer protection against EIH (Green et al., 2011). These results highlight the need for additional research on BP dynamics during and after exercise in recreational cyclists, with a specific focus on the comparison between competitive athletes and recreational or sedentary populations.

Only 20.6 % of participants reported having completed a medical evaluation before starting their recreational cycling practice. Considering the relatively higher prevalence of HTN and other CVD risk factors, this low rate of prior medical evaluation is concerning. The American College of Sports Medicine recommends a personalized approach for pre-participation screening based on the individual's past medical history (CVD, metabolic, or renal disease), current physical activity, and symptoms. Those individuals who are physically active without any CVD, metabolic, or renal disease can continue their exercise activities. However, those with pre-existing CVD or metabolic diseases (including diabetes) and physically inactive might be recommended to obtain a medical evaluation before starting physical activities (Riebe et al., 2015).

This study has several limitations. First, the cross-sectional design precludes causal relationships to be established. Second, the non-probabilistic convenience sampling of our study might have introduced selection bias, limiting our ability to generalize these results. Third, the use of self-reported hypertension might underestimate or overestimate the prevalence of hypertension, particularly in a sample with a low frequency of prior medical evaluations. Fourth, measuring BP at a single time point may not fully reflect the BP profile of participants, and the timing of BP measurement missed the peak BP during exercise for better identification of EIH. Last, due to the nature and design of our study, we were unable to quantify the exercise intensity per participant, limiting our ability to standardize and correlate this with the presence of EIH. On the other hand, the strengths of our study include the relatively large sample size, in addition to the detailed and tailored survey and data collection on key cardiovascular risk factors, prior medical evaluation, and exercise habits in this specific population.

Our findings have important implications for public health and clinical practice. First, strategies are needed to promote regular medical evaluations among recreational cyclists to adequately identify and manage pre-existing cardiovascular risk factors. Second, programs to promote cycling as a healthy physical activity should incorporate education components on the importance of controlling cardiovascular risk factors and the additional benefits of exercise in the management of these conditions (Lavie et al., 2015).

## 5 Conclusions

This study provides a detailed perspective on the prevalence and characteristics of HTN in recreational cyclists. These results emphasize the need to improve preventive medical evaluation practices among recreational cyclists, ensuring the identification and appropriate management of cardiovascular risk factors. Specific interventions for this population may include education programs on the importance of prior medical evaluation and regular BP monitoring, in addition to promoting healthy habits and body weight management. This would help maximize the benefits of cycling while minimizing the risks associated with HTN and other cardiovascular comorbidities.

## CRediT authorship contribution statement

**Bryan Hernandez Nieto:** Writing – original draft, Visualization, Methodology, Investigation, Data curation, Conceptualization. **Manuel Urina-Jassir:** Writing – original draft, Visualization, Methodology, Conceptualization. **Mirary Mantilla-Morron:** Writing – review & editing, Methodology, Investigation, Formal analysis, Data curation, Conceptualization. **Carolina Rosa Charris Cogollo:** Writing – review & editing, Investigation. **Riguey Mercado Marchena:** Writing – review & editing, Investigation. **Daniela Urina-Jassir:** Writing – review & editing, Methodology, Conceptualization. **Manuel Urina-Triana:** Writing – review & editing, Supervision, Methodology, Investigation, Conceptualization. **Miguel Urina-Triana:** Writing – original draft, Visualization, Supervision, Methodology, Investigation, Formal analysis, Data curation, Conceptualization.

## Ethics approval and consent to participate

This study was approved by the Ethics Committee of Fundación del Caribe para la Investigación Biomédica (Acta 296, February 29th, 2024). All participants provided written informed consent to be included in the study.

## Declaration of competing interest

The authors declare that they have no known competing financial interests or personal relationships that could have appeared to influence the work reported in this paper.
